# Robust neutralizing antibodies to SARS-CoV-2 infection persist for months

**DOI:** 10.1126/science.abd7728

**Published:** 2020-10-28

**Authors:** Ania Wajnberg, Fatima Amanat, Adolfo Firpo, Deena R. Altman, Mark J. Bailey, Mayce Mansour, Meagan McMahon, Philip Meade, Damodara Rao Mendu, Kimberly Muellers, Daniel Stadlbauer, Kimberly Stone, Shirin Strohmeier, Viviana Simon, Judith Aberg, David L. Reich, Florian Krammer, Carlos Cordon-Cardo

**Affiliations:** 1 Department of General Internal Medicine, Icahn School of Medicine at Mount Sinai, New York, NY 10029, USA.; 2 Department of Microbiology, Icahn School of Medicine at Mount Sinai, New York, NY 10029, USA.; 3 Graduate School of Biomedical Sciences, Icahn School of Medicine at Mount Sinai, New York, NY 10029, USA.; 4 Clinical Microbiology Laboratory, Department of Pathology, Icahn School of Medicine at Mount Sinai, New York, NY 10029, USA.; 5 Division of Infectious Diseases, Department of Medicine, Icahn School of Medicine at Mount Sinai, New York, NY 10029, USA.; 6 Department of Anesthesiology, Perioperative and Pain Medicine, Icahn School of Medicine at Mount Sinai, New York, NY 10029, USA.

## Abstract

Severe acute respiratory syndrome coronavirus 2 (SARS-CoV-2) has caused a global pandemic with millions infected and more than 1 million fatalities. Questions regarding the robustness, functionality, and longevity of the antibody response to the virus remain unanswered. Here, on the basis of a dataset of 30,082 individuals screened at Mount Sinai Health System in New York City, we report that the vast majority of infected individuals with mild-to-moderate COVID-19 experience robust immunoglobulin G antibody responses against the viral spike protein. We also show that titers are relatively stable for at least a period of about 5 months and that anti-spike binding titers significantly correlate with neutralization of authentic SARS-CoV-2. Our data suggest that more than 90% of seroconverters make detectable neutralizing antibody responses. These titers remain relatively stable for several months after infection.

## SARS-CoV-2 antibodies persist

As the number of daily COVID-19 cases continues to mount worldwide, the nature of the humoral immune response to severe acute respiratory syndrome coronavirus 2 (SARS-CoV-2) remains uncertain. Wajnberg *et al.* used a cohort of more than 30,000 infected individuals with mild to moderate COVID-19 symptoms to determine the robustness and longevity of the anti–SARS-CoV-2 antibody response. They found that neutralizing antibody titers against the SARS-CoV-2 spike protein persisted for at least 5 months after infection. Although continued monitoring of this cohort will be needed to confirm the longevity and potency of this response, these preliminary results suggest that the chance of reinfection may be lower than is currently feared.

*Science*, this issue p. 1227

## Teaser

Antibodies generated after mild to moderate SARS-CoV-2 infection are neutralizing and relatively stable for at least 5 months.

Severe acute respiratory syndrome coronavirus 2 (SARS-CoV-2) has infected millions of individuals globally and, as of October 2020, has led to the death of >1 million individuals. Although the antibody responses in severe COVID-19 cases have been relatively well characterized (*1*, *2*), assessing the response in mild and asymptomatic cases is of great importance because they constitute most infections. It will be critical to understand the robustness of the antibody response in these mild cases, including its longevity and functionality, so as to inform serosurveys and to determine levels and duration of antibody titers that may be protective against reinfection (*3*).

Antibodies to SARS-CoV-2 can target many of its encoded proteins, including structural and nonstructural antigens. Thus far, two structural proteins have been used as target antigens for serological assays. One is the abundant nucleoprotein (NP), which is found inside the virus or inside infected cells. However, because of the biological function of NP and because it is shielded from antibodies by viral or cellular membranes, it is unlikely that NP antibodies can directly neutralize SARS-CoV-2. The second structural protein often used as a target for characterizing the immune response to SARS-CoV-2 is the spike protein. The spike is a large trimeric glycoprotein that contains the receptor binding domain, which the virus uses to dock to its cellular receptor, angiotensin-converting enzyme 2, and for fusion of viral and cellular membranes (*4*, *5*). It is known from other coronaviruses—and it holds true for SARS-CoV-2—that the spike is the main, and potentially the only, target for neutralizing antibodies (*6*). Therefore, the assay used in this study to characterize the antibody response to SARS-CoV-2 is based on the trimerized, stabilized ectodomain of the spike protein (*7*). An enzyme-linked immunosorbent assay (ELISA) initially developed in early 2020 has been extensively used in research (*7*–*10*). This so-called Mount Sinai ELISA has high sensitivity (92.5%) and specificity (100%), as determined with an initial validation panel of samples (table S1). Furthermore, it has a positive predictive value (PPV) of 100%, with a negative predictive value (NPV) of 99.6%.

**Fig. 1:**
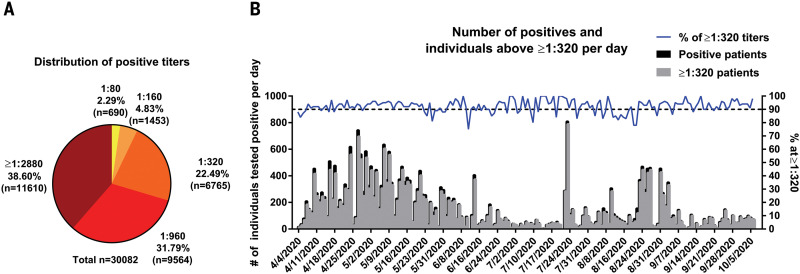
SARS-CoV-2 spike antibody titers in 30,082 individuals. (**A**) The percentage of individuals with antibody titers of 1:80 (low), 1:160 (low), 1:320 (moderate), 1:960 (high), and ≥1:2880 (high). (**B**) Absolute numbers of individuals testing positive and percent of individuals with titers of 1:320 over time. Testing of each sample was performed once in a Clinical Laboratory Improvement Amendments (CLIA)–certified laboratory using an assay that received emergency use authorization (EUA) from the U.S. Food and Drug Administration (FDA).

In March 2020, the Mount Sinai Health System began screening individuals for antibodies to SARS-CoV-2 to recruit volunteers as donors for convalescent plasma therapy (*11*). Screened patients had either polymerase chain reaction (PCR)–confirmed SARS-CoV-2 infections or suspected disease, which is defined as being told by a physician that symptoms may be related to SARS-CoV-2 or exposure to someone with confirmed SARS-CoV-2 infection. Most of the symptomatic patients who were screened experienced mild-to-moderate disease, with <5% requiring emergency department evaluation or hospitalization. Mount Sinai also offered the antibody test to all employees in its health system on a voluntary basis. By 6 October 2020, Mount Sinai had screened 72,401 individuals, with a total of 30,082 individuals testing positive (defined as detectable antibodies to the spike protein at a titer of 1:80 or higher) and 42,319 testing negative. The clinical laboratory ELISA set up results in discrete titers of either 1:80, 1:160, 1:320, 1:960, or ≥1:2880. Titers of 1:80 and 1:160 were categorized as low titers, 1:320 as moderate, and 1:960 and ≥1:2880 as high titers. For plasma therapy, titers of 1:320 or higher were initially deemed eligible. Of the 30,082 positive samples, 690 (2.29%) had a titer of 1:80; 1453 (4.83%), 1:160; 6765 (22.49%), 1:320; 9564 (31.79%), 1:960; and 11,610 (38.60%), ≥1:2880 (Fig. 1). Thus, we conclude that the vast majority of positive individuals have moderate-to-high titers of anti-spike antibodies. Of course, the argument could be made that we could be missing a number of individuals who had been infected with SARS-CoV-2 and did not produce antibodies, given that many individuals included in our dataset had never been tested by a nucleic acid amplification test for the virus. An earlier analysis performed with a smaller subset of 568 PCR-confirmed individuals using the same ELISA showed that >99% developed an anti-spike antibody response (*8*). In a later dataset of 2347 patients who self-reported positive PCR, 95% had positive antibody titers, which indicates that we did not miss large numbers of patients and confirms our prior sensitivity findings. Thus, the rate of individuals who do not seroconvert after SARS-CoV-2 infection is low, although such individuals may exist, and the majority of responders mount titers of 1:320 or higher.

**Fig. 2:**
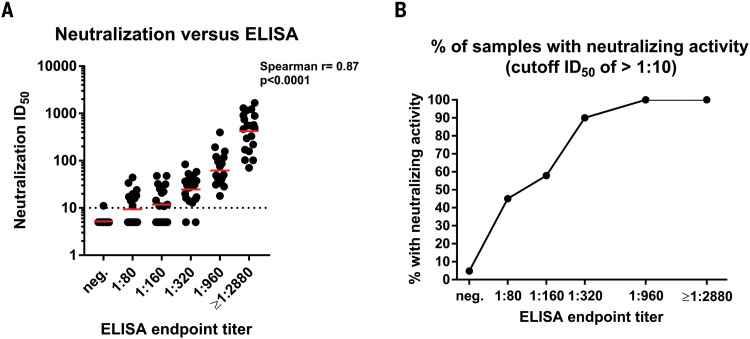
Neutralizing activity of serum samples in relation to ELISA titers. (**A**) A correlation analysis between ELISA titers on the *x* axis and neutralization titers in a microneutralization assay on the *y* axis. The Spearman ρ was determined. Red bars indicate the geometric mean. (**B**) The proportion of sera that exert any neutralizing activity in each of the ELISA titer categories. Testing was performed once, using an FDA EUA ELISA in a CLIA laboratory, or twice, following a standardized neutralization protocol.

Determining the neutralizing effects of SARS-CoV-2 spike antibodies is critical to understanding possible protective effects of the immune response. Therefore, we performed a well-established quantitative microneutralization assay (*12*) based on authentic SARS-CoV-2 with 120 samples of known ELISA titers ranging from negative to ≥1:2880. Neutralization titers significantly correlated (Spearman ρ = 0.87, *P* < 0.0001) with spike-binding titers (Fig. 2A). Although there was some variability, sera with 1:320, 1:960, and ≥1:2880 ELISA titers had geometric mean 50% inhibitory dilutions (ID_50_) of about 1:30, 1:75, and 1:550, respectively. If any and all neutralizing activity above background is considered, then ~50% of sera in the 1:80 to 1:160 titer range, 90% in the 1:320 range, and all sera in the 1:960 to ≥1:2880 range had neutralizing activity (Fig. 2B). Only one of the negative samples showed activity slightly above background, which was potentially an ELISA false negative.

**Fig. 3:**
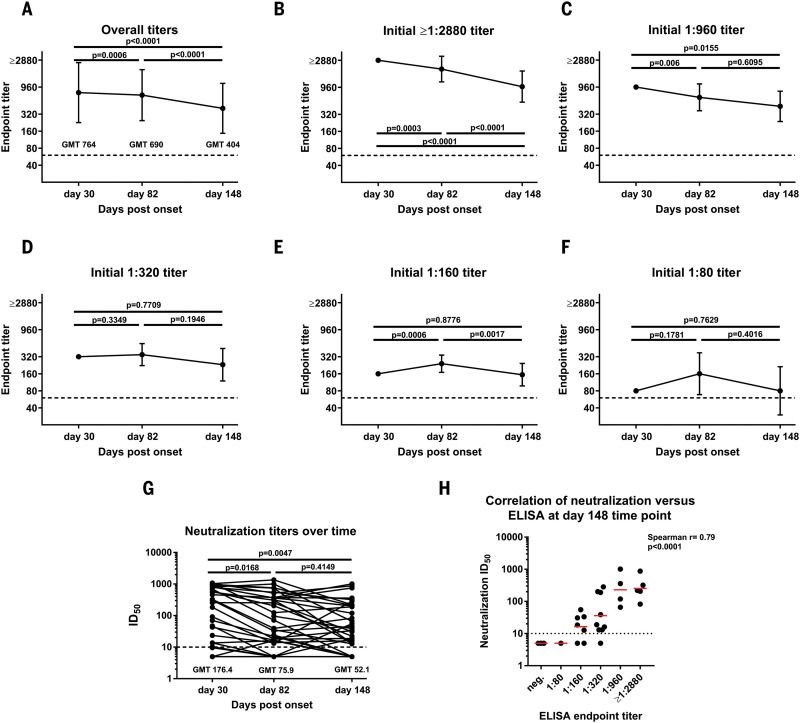
Antibody titer stability over time. (**A**) Titers of 121 volunteers whose blood was initially drawn ~30 days after COVID-19 symptom onset and who were then recalled for additional blood draws at ~82 days and 148 days after symptom onset. (**B** to **F**) The same data as in (A), but stratified by the initial (day 30) titer. Titers are graphed as geometric mean titers (GMT) with geometric standard error. (**G**) Neutralization titers of 36 individuals over time. A paired one-way analysis of variance corrected for multiple comparison was used to determine statistical significance. (**H**) A correlation analysis between ELISA titers on the *x* axis and neutralization titers in a microneutralization assay on the *y* axis at day 148. Red bars indicate the geometric mean. The Spearman ρ was determined. Testing was performed once, using an FDA EUA ELISA in a CLIA-certified laboratory, or twice, following a standardized neutralization protocol.

Another important question is longevity of the antibody response to the spike. To assess the medium-range stability of serum antibody titers against the spike protein, we recalled 121 plasma donors at a variety of titer levels who had initially been screened at around day 30 after symptom onset for two additional time points. The mean interval between the initial titer measurement and the second was 52 days (range: 33 to 67 days). This set the second time point at a mean of 82 days after symptom onset (range: 52 to 104 days) and the third time point at 148 days after symptom onset (range: 113 to 186 days). In comparing overall titers, we observed a slight drop from a geometric mean titer (GMT) of 764 to a GMT of 690 from the first to the second time point and another drop to a GMT of 404 for the last time point (Fig. 3A). In the higher titer range of ≥1:2880 and 1:960, we also observed a slow decline in titer over time (Fig. 3, B and C). Unexpectedly, but in agreement with earlier observations that seroconversion in mild COVID-19 cases might take a longer time to mount (*8*), we saw an increase in individuals who had an initial titer of 1:320, 1:160, or 1:80 (Fig. 3, D to F) from day 30 to day 82. Titers in these groups declined to about day 30 levels on day 148. Notably, one individual in the initial 1:80 group dropped from a 1:80 titer to being negative at the day 82 time point, and two others lost reactivity at the day 148 time point, indicating that very low initial titers might drop to undetectable levels over time. Neutralizing antibody titers followed titers measured by ELISA (Fig. 3G), and a good correlation between neutralization and ELISA titers was still observed on day 148 (Fig. 3H). The initial serum antibody titer was likely produced by plasmablasts, and plasmablast-derived antibodies peak 2 to 3 weeks after symptom onset. Given an immunoglobulin G half-life of ~21 days, the sustained antibody titers observed here over time are likely produced by long-lived plasma cells in the bone marrow. Note that our observations contrast with a recent report that found waning titers at 8 weeks after virus infection (*13*). Especially in asymptomatic cases, antibody responses disappeared after 8 weeks in 40% of individuals in that study. However, the antibodies measured in that paper targeted the NP plus a single linear spike epitope. The same paper also reported relatively stable (slightly declining) neutralizing antibody titers, which shows much higher concordance with our present findings. Thus, the stability of the antibody response over time may also depend on the target antigen.

Correlates of protection have been established for many different viral infections. These correlates are usually based on a specific level of antibody acquired through vaccination or natural infection that substantially reduces the risk of (re)infection. One example is the hemagglutination inhibition titer for the influenza virus, where a 1:40 titer reduces the risk of getting infected by 50% (*14*). Similar titers have been established for the measles virus (an ID_50_ titer of 1:120), hepatitis A virus, hepatitis B virus, and many others (*15*). These titers have facilitated vaccine development considerably. For some viruses and vaccines, the kinetics of the antibody response is also known, allowing for an accurate prediction of how long protection will last (*16*).

It is still unclear whether infection with SARS-CoV-2 in humans protects from reinfection and, if it does, for how long. We know from work with common human coronaviruses that neutralizing antibodies are induced and that these antibodies can last for years and provide protection from reinfection or, in the event of reinfection, attenuate disease (*17*). Furthermore, we now know from nonhuman primate models that infection with SARS-CoV-2 does protect from reinfection for at least some time (*18*, *19*). We also know that transferring serum of convalescent animals or neutralizing monoclonal antibodies to naïve animals can be protective and reduces virus replication significantly (*20*, *21*). Finally, vaccine-induced neutralizing antibody titers have been established as a correlate of protection in nonhuman primates (*22*). Notably, these vaccine-induced titers were relatively low and in the lower range of the titers observed in this study. Our data reveal that individuals who have recovered from mild COVID-19 experience relatively robust antibody responses to the spike protein, which correlate significantly with neutralization of authentic SARS-CoV-2 virus. Furthermore, the vast majority of individuals with antibody titers of 1:320 or higher show neutralizing activity in their serum. We also find stable antibody titers over a period of at least 3 months and only modest declines at the 5-month time point, which is consistent with data for the human coronaviruses SARS-CoV-1 and Middle East respiratory syndrome–related coronavirus (MERS-CoV) (*17*). We plan to follow this cohort over longer intervals of time. Although we cannot provide conclusive evidence that these antibody responses protect from reinfection, we believe it is very likely that they will decrease the odds ratio of reinfection and may attenuate disease in the case of breakthrough infection. We believe that it is imperative to swiftly perform studies to investigate and establish a correlate of protection from SARS-CoV-2 infection. A correlate of protection, combined with a better understanding of antibody kinetics to the spike protein, would inform policy regarding the COVID-19 pandemic and would be beneficial to vaccine development efforts.

## Supplementary Materials

science.sciencemag.org/content/370/6521/1227/suppl/DC1 Materials and Methods Table S1 References MDAR Reproducibility Checklist

## Appendix

View/request a protocol for this paper from *Bio-protocol*.
